# Editorial Comment to Rare gastrointestinal stromal tumor in an ileal conduit detected by recurrent massive bleeding from the stoma

**DOI:** 10.1002/iju5.12198

**Published:** 2020-08-08

**Authors:** Shinya Yamamoto

**Affiliations:** ^1^ Department of Genitourinary Oncology Japanese Foundation for Cancer Research Cancer Institute Hospital Tokyo Japan

This is a very valuable report of a gastrointestinal stromal tumor (GIST) detected in an ileal conduit that recurred 19 years after radical cystectomy. The GIST was found on computed tomography scan due to bleeding from the stoma and was resected by laparotomy. The patient has had no recurrence for 4 years.^1^

The occurrence of secondary tumor associated with different forms of urinary diversion is rare, and the incidence is approximately 0.18% in the literature. Kälble *et al*. analyzed 32 secondary tumors occurring in 17 758 urinary diversions performed between 1970 and 2007. They reported that, of the 32 cases, the secondary tumor risk of ureterosigmoidostomy (2.58%) was the highest, and the second highest was cystoplasty (1.58%). The secondary tumor risk of ileal neobladder (0.05%) and ileal conduit (0.02%), which comprise the majority of all urinary diversions, is extremely low. Kälble *et al*. discussed the difference in secondary tumor risk of various urinary diversions, and speculated that it might result from the difference of colon *vs* ileum rather than the urinary diversion itself. In terms of pathological type, adenocarcinomas are reported to be the most common, followed by adenomas.^2^ The pathological diagnosis of this case was GIST, which is very rare. The occurrence of GIST associated with different forms of urinary diversion is very rare, and there is only one other case in the worldwide literature. The previous case was GIST arisen from the ileal neobladder,^3^ and the case in this report is the first case of GIST in the ileal conduit.

With regard to treatment, surgery was performed for both cases. Surgery is necessary for secondary tumor without distant metastasis in urinary diversion, because tumor biopsy can be difficult.

The secondary tumor latency period after urinary diversion varies by urinary diversion and tumor histology, although the secondary tumor in this case was found 19 years postoperatively. The authors recommend regular checks after urinary diversion to monitor the development of secondary tumors – how long should this follow‐up be in patients with urinary diversion?

The spread of robot‐assisted radical cystectomy and the accompanying skill improvement is expected to result in an increase in long‐term survivors after urinary diversion in the near future. As such, this case report will be of significant importance to urologists because an increase in secondary tumors after urinary diversion can be expected. Careful monitoring for secondary tumors after urinary diversion is thus essential.

## Conflict of interest

The author declares no conflict of interest.
